# Evolution of SO_2_ and NOx Emissions from Several Large Combustion Plants in Europe during 2005–2015

**DOI:** 10.3390/ijerph17103630

**Published:** 2020-05-21

**Authors:** Daniel-Eduard Constantin, Corina Bocăneala, Mirela Voiculescu, Adrian Roşu, Alexis Merlaud, Michel Van Roozendael, Puiu Lucian Georgescu

**Affiliations:** 1 Faculty of Sciences and Environment, European Centre of Excellence for the Environment, “Dunarea de Jos” University of Galati, Domneasca Street, no. 111, 800201 Galati, Romania; Corina.Bocaneala@ugal.ro (C.B.); mirela.voiculescu@ugal.ro (M.V.); rosu_adrian_90@yahoo.ro (A.R.); lucian.georgescu@ugal.ro (P.L.G.); 2 Royal Belgian Institute for Space Aeronomy (BIRA-IASB), Ringlaan-3-Avenue Circulaire, B-1180 Brussels, Belgium; alexis.merlaud@aeronomie.be (A.M.); michel.vanroozendael@aeronomie.be (M.V.R.)

**Keywords:** Large Combustion Plant (LCP), NOx, SO_2_, emissions, Ozone Monitoring Instrument

## Abstract

The aim of this paper is to investigate the evolution of SO_2_ and NOx emissions of ten very large combustion plants (LCPs >500 MW) located in the European Union (EU) during 2005–2015. The evolution of NOx and SO_2_ emissions were analyzed against the EU Directives in force during 2005–2015. The investigation was performed using space-borne observations and estimated emissions collected from the EEA (European Environment Agency) inventory of air pollutant emissions. The power plants were chosen according to their capacity and emissions, located in various parts of Europe, to give an overall picture of atmospheric pollution with NOx and SO_2_ associated with the activity of very large LCPs in Europe. Satellite observations from OMI (Ozone Monitoring Instrument) are compared with calculated emissions in order to assess whether satellite observations can be used to monitor air quality, as a standard procedure, by governmental or nongovernmental institutions. Our results show that both space observations and estimated emissions of NOx and SO_2_ atmospheric content have a descending trend until 2010, complying with the EU Directives. The financial and economic crisis during 2007–2009 played an important role in reducing emissions.

## 1. Introduction

Atmospheric pollution is one of the largest environmental health risks in Europe, severely affecting human health and causing more than 400,000 premature deaths each year [1]. More than 50% of the SO_2_ and NOx emissions in Europe are associated with the production and distribution of fossil fuels [2]. It is well known that the emissions from thermal processes of fossil fuels have negative effects on human health and the environment. High levels of NO_2_ can lead to cardiovascular dysfunctions and respiratory problems such as cold, bronchitis, asthma, and lung cancer [3,4] while the effects of sulfur dioxide on human body consists of irritation of the airways, coughing, shortness of breath and a sensation of tightening around the chest [5]. Large quantities of SO_2_ and NO_2_ can lead to acid rains and disturbances in the functioning and structure of ecosystems, e.g., the acidification of soils and waters [6].

Large combustion plants (LCPs) are combustion plants with a total rated thermal input equal to or greater than 50 MW and have an important contribution to the air quality degradation due to anthropogenic pollutant emissions in the atmosphere [7]. EU legislation has set specific emission limit values for NOx, SO_2,_ and dust emissions from installations with a rated thermal input of ≥50 MW. LCPs use fossil fuels to produce thermal or electric energy, resulting in residues and waste products (including emissions) that affect the quality of all environmental components, with a specific and clear impact on the atmospheric composition. Large amounts of trace gases (NOx, SO_2_, CO_2_) and particulate matter (PM) are released in the atmosphere when LCPs are operational. 

The first EU policy related to emissions control dates from the 1980s. Between 2005 and 2015 the main legislation related to the power plants was governed by: the LCP Directive (Directive 2001/80/EC) [8], the NEC (National Emission Ceilings) Directive (2001/81/EC) [9], the IPPC Directive (Directive 2008/1/EC) [10] and the Industrial Emissions Directive (IED, Directive 2010/75/EU) [11]. The Directive 2001/80/EC set up limitations for sulfur dioxide (SO_2_), nitrogen oxides (NOx), and dust emissions from large combustion plants. The NEC Directive introduced upper limits of national emissions for five important air pollutants: nitrogen oxides (NOx), non-methane volatile organic compounds (NMVOCs), sulfur dioxide (SO_2_), ammonia (NH_3_), and fine particulate matter (PM_2.5_). The measures of the LCP Directive were binding from 2008 until December 2015. As of 2010, according to the NEC Directive, all the EU states are required to meet their emission ceilings. Also, since 2010 the IED Directive imposed stack emission control and thresholds for each trace gas or PM, in order to reduce emissions. Note that the LCP and NEC Directives refer to reduction plans for each EU country at a national level by specifying a maximum total amount of emissions per year, without imposing specific measures for a certain plant. The Directive 2010/75/EU (IED), which replaced Directive 2001/80/EC (LCPD), comes with more restrictive measures, by constraining emissions locally, at the point where these leave the installation. However, exemptions from regulations for many EU countries (the so-called “Transitional National Plan”) could prolong the implementation of the reduction measures until 30 June, 2020 [11]. The restrictive measures of the LCP and NEC Directives are less tight than the new IED Directive. Consequently, the EU countries were free to manage the emission ceiling by closing certain power plants or reducing the number of operating hours [8,9].

Here we focus on two trace gases, NO_2_ and SO_2_, which are released into the atmosphere during thermal processes of fossil fuels when LCPs are operational. One of the purposes of this paper is to test whether satellite measurements of NO_2_ and SO_2_ can be used as proxies for NO_2_ or SO_2_ emissions over different areas of the Earth’s surface, thus replacing or supplementing the estimated emission calculations. 

Satellite-based observations of atmospheric parameters have many applications, ranging from climate change monitoring to trace gas observations [12,13]. Moreover, space observations can provide self-consistent information about the evolution of NO_2_ and SO_2_ on a continuous-time basis, based on daily global coverage [13]. Spectroscopic measurements of NO_2_ and SO_2_ using UV-Vis DOAS space observations have been available since July 1995, when the Global Ozone Monitoring Experiment (GOME-1) was launched into space onboard ESA’s 2nd European Remote Sensing Satellite (ERS-2) [14,15]. Space-based DOAS observations of tropospheric NO_2_ and SO_2_ have, since then, become a very useful tool for monitoring of emissions from different sources at a global or regional level [16,17,18]. One such tool is the Ozone Monitoring Instrument (OMI), which is a space UV-Vis spectrometer based onboard of the AURA satellite, used for NO_2_, SO_2,_ and other trace gas observations [19]. Space observation can be used for estimation of the quantity of NO_2_ and SO_2_ from various sectors of activity, including energy production [20,21,22,23].

Trends of regional NO_2_ or SO_2_ reported by satellite observations are different. For instance, a decrease of SO_2_ and NO_2_ emissions are reported for China [24,25], USA [26,27], and Europe [28], while increased emissions are observed for India [29]. Emissions from LCPs constitute a large proportion of total anthropogenic emissions. In 2015, LCP emissions of SO_2_ and NOx made up 44% and 14%, respectively, of total EU-28 emissions of these pollutants [2]. EU imposed specific emission limit values on emissions of NOx, SO_2,_ and dust from plants with a rated thermal input equal to or greater than 50 MW. Also, an important role in the emission reduction was induced by the deindustrialization of many European countries; the most affected being the countries of Eastern Europe.

**Figure 1:**
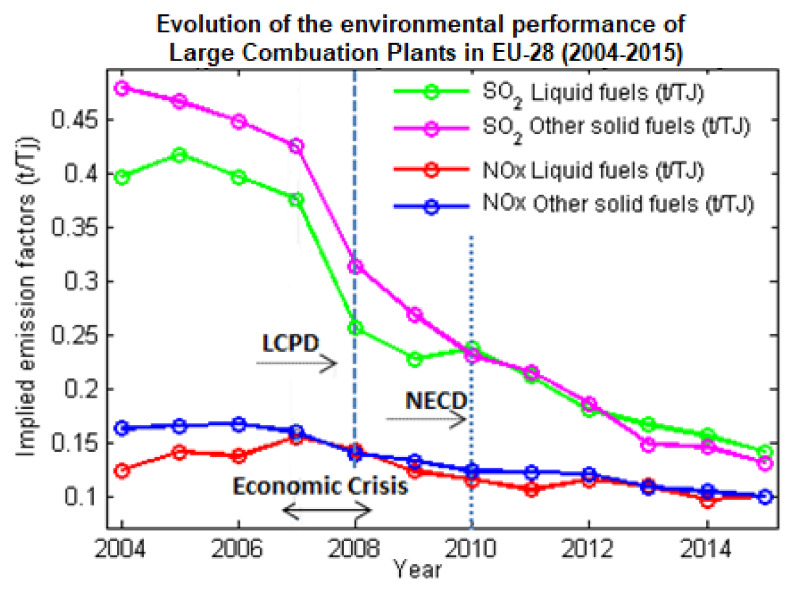
Evolution of the environmental performance of large combustion plants in the EU-28, expressed as implied emission factors for SO_2_ and NOx by fuel type.

Figure 1 shows the evolution of the environmental performance of LCPs during 2004–2015 in the EU-28, expressed as implied emission factors for SO_2_ and NOx by fuel type. The implied emission factor is the ratio between emissions and fuel consumption. The implied emission factor for the NOx and SO_2_ pollutants decreased significantly between 2005 and 2015 for large combustion plants of different sizes [30]. However, according to the 2015 and 2017 indicator assessment [31,32] presented by the EEA, the emission reductions cannot be linked only to environmental policies implementation, but to other factors as well, e.g., broader economic and societal changes, economic conditions, international fuel prices, industry initiatives, etc. [30]. Singhal in 2019 presented a comprehensive study regarding the emissions reduction from LCPs in the European policies context; he concluded that the LCP Directive was an effective instrument in pollution abatement at the stack-level [33]. Also, Meyer and Pac (2017) [34] discussed the consequences of the LCP Directive over the 1585 EU’s large combustion plants. 

The aim of this study is to analyze the evolution of NO_2_ and SO_2_ using space observations of the OMI instrument and EEA reported emissions for ten large power plants located in the EU, in order to assess the effect of EU standards and regulations implementation. This paper is organized as follows. Data and methodology are described in Section 2, results and discussions are presented in Section 3, while Section 4 is dedicated to conclusions. 

## 2. Materials and Methods 

**Figure 2:**
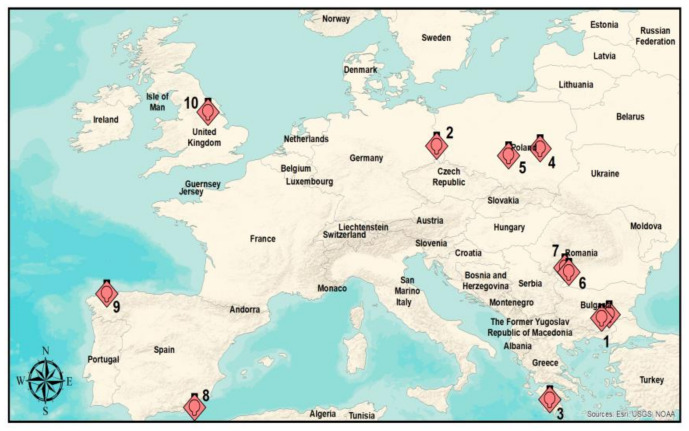
The spatial distribution of LCPs; these are identified by the corresponding numbers in Table 1.

Ten European power plants were selected, considering their capacity and quantity of NOx and SO_2_ emissions (https://www.eea.europa.eu/data-and-maps/data/lcp-9). The LCPs are located in various parts of the European continent (Figure 2) and are considered as very large combustion plants (>500 MW). Location (i.e., latitude and longitude) and the annual average of NOx and SO_2_ emissions level for the selected power plants are presented in Table 1.

The NO_2_ and SO_2_ satellite data were provided by the OMI space instrument as grid-averaged columnar amounts. OMI is a nadir-viewing UV-Vis spectrometer that measures atmospheric trace gases and aerosols. It provides daily global observations at a resolution of 13 km × 24 km. NO_2_ was gathered from the Tropospheric Emission Monitoring Internet Service (TEMIS) database (http://temis.nl/airpollution/NO2.html). OMI monthly-mean tropospheric NO_2_ columns were based on the Dutch OMI NO_2_ version 2.0 product, which is a post-processing data set performed at Koninklijk Nederlands Meteorologisch Instituut (KNMI) [35]. We used the ESRI grid format with a cell size 0.125 degree. Note that the OMI sensor can provide only information about NO_2_; in the case of intercomparisons, the NOx emissions estimated from the ground will be expressed as NO_2_ equivalent. The SO_2_ data were obtained from the NASA Geospatial Interactive Online Visualization ANd aNalysis Infrastructure (Giovanni) interface, which is a remote-sensing and model data Web-based analysis and visualization system developed by the Goddard Earth Sciences Data and Information Services Center (GES DISC) [36]. We used the SO_2_ Column Amount (Planetary Boundary Layer) OMSO2e v003 [37] available on https://giovanni.gsfc.nasa.gov/giovanni/. This is a Level-3 Aura/OMI global SO_2_ data product, based on grids (0.25-degree Latitude/Longitude grids) containing one observation of total column density of SO_2_ in the planetary boundary, derived from an improved band residual difference algorithm (BRD) [38,39]. The NO_2_ and SO_2_ columnar amount, within a grid cell of 0.25 degrees centered on each LCP center, were considered for this study. Data regarding NOx and SO_2_ emissions between 2005 and 2015 are obtained from the European Environment Agency online database.

## 3. Results

This section presents the evolution of SO_2_ and NO_2_ emissions reduction as observed from space or derived from the ground in the context of main industrial emissions directives which governed the period 2005–2015, especially the LCP and NEC Directives. The implications and main drivers that could lead to decreases or increases in emissions are introduced in this section.

**Figure 3:**
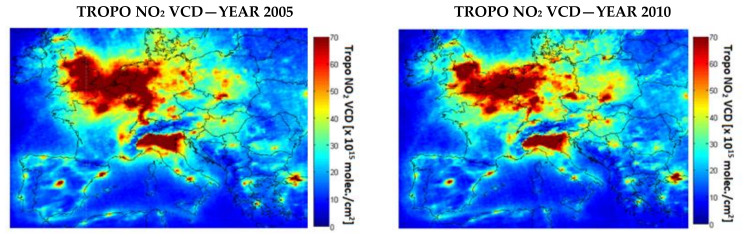
Annual tropospheric NO_2_ VCD based on OMI observations for 2005 vs. 2010.

**Figure 4:**
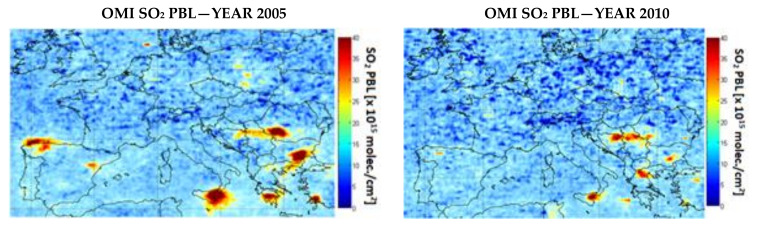
Annual tropospheric SO_2_ PBL based on OMI observations for 2005 vs. 2010.

Figure 3 and Figure 4 present maps of NO_2_ and SO_2_ tropospheric amounts observed by OMI, over Europe during 2005 and 2010. We show 2005 because this is the first full year of OMI measurements and 2010 because this was the milestone year of the NEC Directive. Hotspots can be clearly associated with the power plants, e.g., Greece, Bulgaria, and Romania. The NO_2_ decrease over the selected power plant is visible from OMI (Figure 3) but the most important drop is visible in the case of SO_2_ (Figure 4).

### 3.1. Nitrogen Oxides

**Figure 5:**
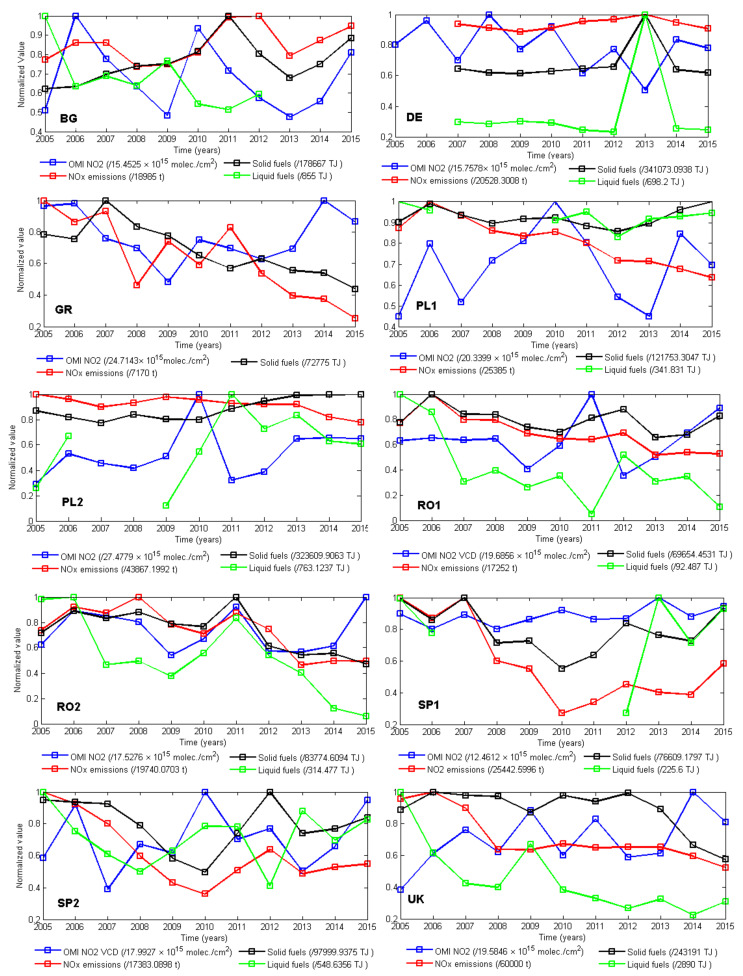
Time series of normalized NO_2_ during 2005–2015 using data from satellite instruments (blue) and emissions (red) for each station, together with the reported solid fuel (black) and liquid fuel consumption (green).

Figure 5 shows the NO_2_ evolution for each power plant, during 2005–2015, resulting from OMI measurements of the tropospheric NO_2_VCDs and from estimated emissions. The latter are reported by each EU country to the EEA. Plots include the consumption of liquid and solid fuels per LCP. All measurements are normalized to their maximum. Expectedly, emission calculations follow roughly the trend of solid/liquid consumption, since the calculation of the former are based on the latter. 

Almost all reported emissions of NO_2_ showed a decreasing trend, except plants in BG and DE, while OMI space observations show an increasing trend for all, except plants in BG and GR. Note that one cannot expect a one-to-one correspondence between the reported emissions and space observations. On the one hand, differences between emissions and OMI stem from the fact that the satellite instrument sees a large area (13 km × 24 km), thus measurements contain background NO_2_ and the NO_2_ released by surrounding local/traffic sources. Secondly, satellite results are subject to various assumptions related to the atmospheric mass factor (AMF) calculations. The emissions, on the other hand, are based on calculations based on the quantity and nature of fossil fuel. The type and quality of fuel may influence the quantity of emissions, the correlation between the quantity of calculated emissions, and the nature of the fossil fuel (solid or liquid) which could give the main fuel used during the thermal processes. The discrepancies may also be due to the fact that OMI soundings are done at a specific time (i.e., the overpass time), which is associated with various phases of the diurnal variation of NO_2_, depending on the geographical location of the station.

OMI measurements and estimated emissions correlate fairly well (R >0.5) for a few power plants: BG, RO2, and SP1. However, for the other plants, there is practically no correlation since minima and maxima in the two-time series are even opposing (e.g., SP2, DE, and GR). A clear peak is seen in the NO_2_ satellite time series around 2010–2011, when the winter was unusually cold in Europe [40], for all stations except stations GR, SP1, and the UK. The first are the southernmost ones, which probably were less affected by the low temperatures, while the UK had a different climate compared to the continent. The increased NO_2_ concentration may be the result of a combination of the increased request for heating and a higher lifetime of NO_2_, of the order of days, when temperatures are low. An important characteristic of the NO_2_ evolution, very well highlighted in OMI space observations, was the global financial and economic crisis during 2007–2009 [26,41,42,43]. Considering the influence of the financial and economic crisis (2007–2009) on the emissions, the end of recessions could correspond to the emissions increase in 2010 and after this year. 

**Figure 6:**
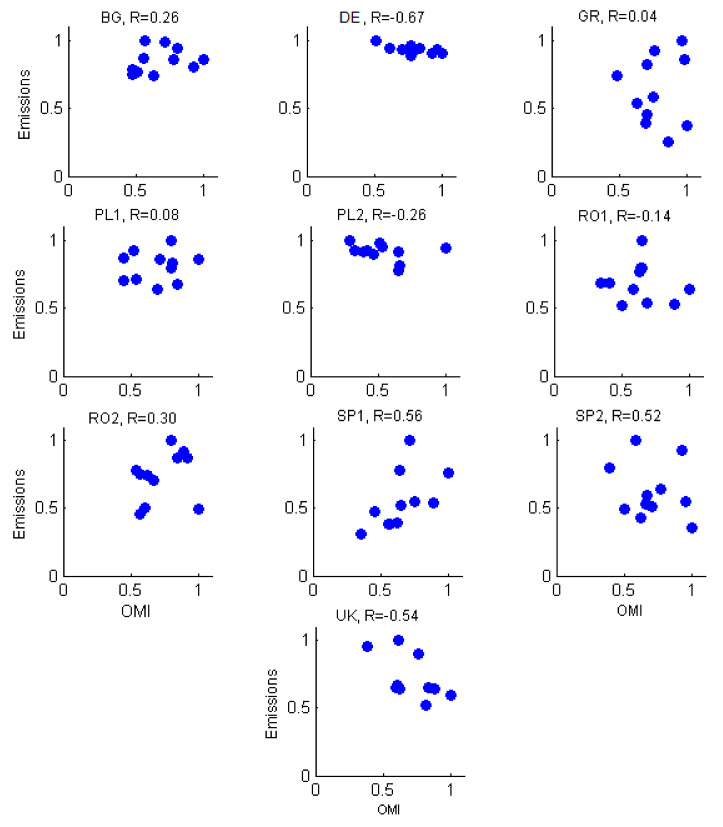
Scatter plots of OMI measurements vs. reported emission for NO_2_ (normalized values).

Scatter plots of OMI measurements against the reported emission for NO_2_ amount are shown in Figure 6, for all plants. Satellite measurements seemed to be a good indicator for emission variability only for some LCPs: EP1, EP2, BG, and RO2, for which correlation coefficients were positive (R = 0.56, 0.52, 0.26, 0.32). For other plants, the emissions varied in a completely different manner than the satellite measurements of tropoVCDs. Low emissions corresponded to high values of satellite VCDs of NO_2_ and vice versa. Large negative correlations between the two datasets were seen, e.g., in the UK (R = −0.54) or DE (R = −0.67). Indeed, as mentioned before, satellite data will inevitably contain also the background NO_2_ resulting from all sources in an area of about 300 km^2^. VCDs also respond to background conditions (higher or lower temperatures, wind effect, urban agglomeration), while emissions relate only to fuel consumption. For LCPs where OMI showed an increasing trend while the calculated emissions show the opposite, the explanation could be the fact that the calculated emissions from LCP do not include the nearby emissions, as urban and traffic emissions, or other industries; the latter are however included in space observations. This may be the case since Figure 5 shows that towards the end of the selected period, most emissions decreased, while tropospheric VCDs increased. However, the very large differences seen for other stations cannot be explained.

### 3.2. Sulfur Dioxide

**Figure 7:**
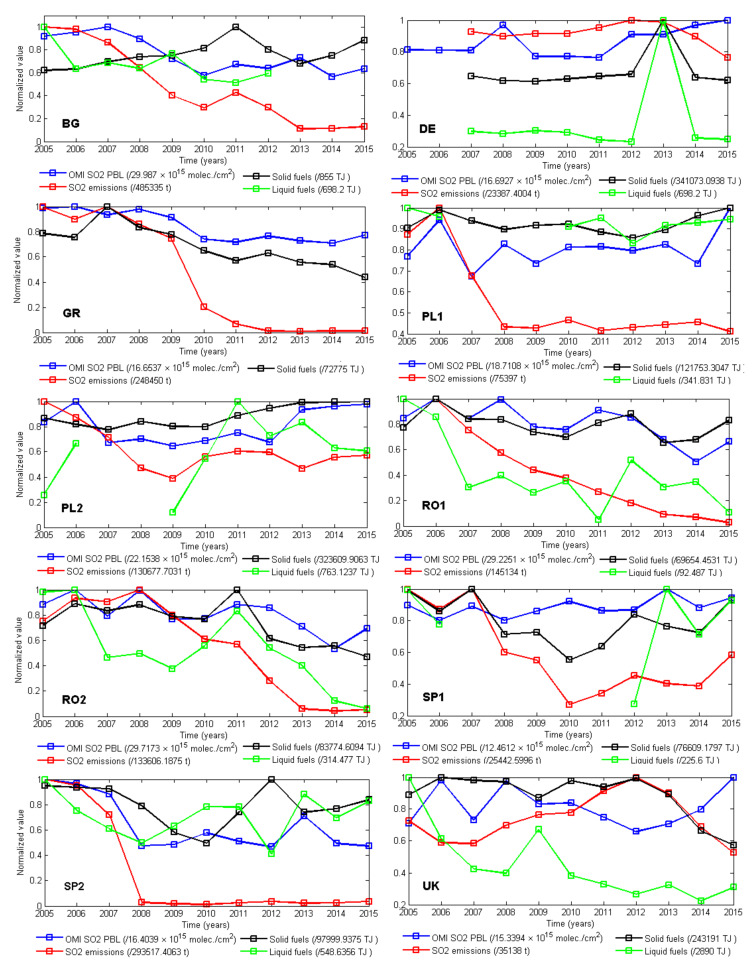
Time series of normalized SO_2_ during 2005–2015 using data from satellite instruments (blue) and emissions (red), together with the reported solid fuel (black) and liquid fuel consumption (green).

The SO_2_ emissions of power plants in Europe have drastically decreased over the last 20 years. Figure 7 shows that during 2005–2015, the observed SO_2_ observed by OMI (blue line) has no trend (as in PL1, PL2, SP1, and the UK) or is slightly decreasing (BG, GR, RO1, EO2, and SP2). The emissions (red line), on the other hand, have a clear decreasing trend for most stations, except the UK, with a maximum in 2012, and DE, where the highest emissions were reported also in 2012. 

Similar to NO_2_, we do not aim to compare satellite measurements, emission, and fuel consumption, but their annual variation (this is also why we use normalized values). The similarities between satellite data and emissions are better for SO_2_ than for NO_2_. Some discrepancies between the two-time series were seen for the UK, PL1, or DE. Emissions decreased more abruptly than to VCD measurements in GR, PL1, RO2, RO1, SP2, and SP1. Even if the trend was the same, differences between the relative variations of VCDs and emissions were still high (e.g., GR, PL1, RO1, and SP2) (Figure 7). Important factors to be considered are (1) the overpass time of the space sensor over the emission source and, as in the case of NO_2_, and (2) the contribution of other sources to the SO_2_ amount measured by satellite. Except for the two plants from Spain (SP1 and SP2), SO_2_ emissions decreased, on average, after 2010. Satellite measurements confirmed the SO_2_ decrease, especially for BG and GR. The descending trend of reported SO_2_ emissions was higher compared to the descending trend of SO_2_ observed from space. The correlation between the SO_2_ content observed from space and the type of fossil fuel may indicate whether the main used fuel is liquid or solid. 

The large difference between the trend of reported emissions and space observations over the power plants from Greece, Romania, and Spain can be explained by the use of improved SO_2_ filter systems, e.g., the case of Romanian LCPs [44,45].

**Figure 8:**
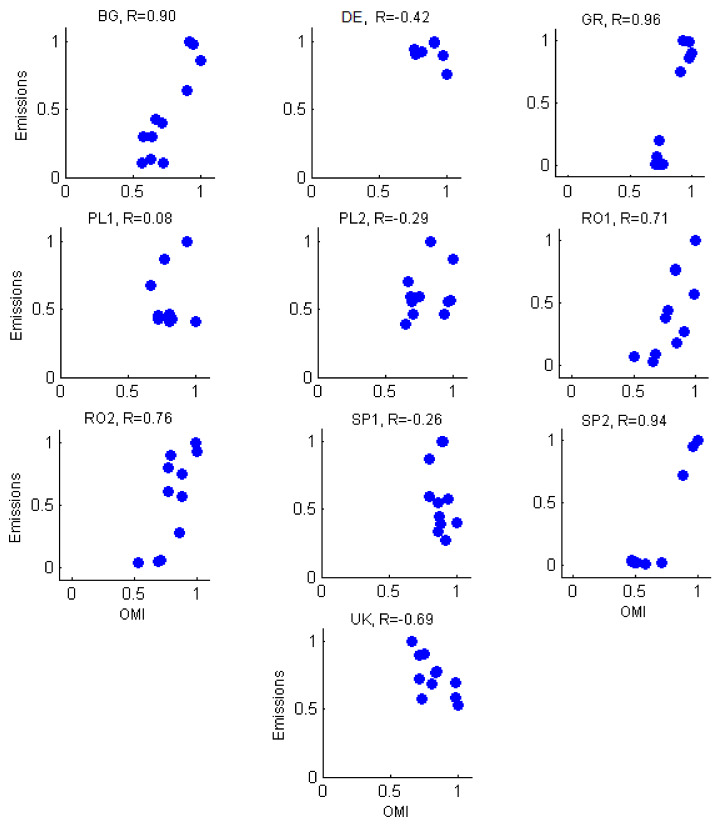
Scatter plots of OMI measurements vs. emissions for SO_2_ (normalized values).

Figure 8 confirms that SO_2_ satellite measurements correlated better to the corresponding emissions compared to NO_2_ for BG, GR, PL2, RO1, RO2, and SP2. The variation of SO_2_ emissions of the UK, DE, PL1, and SP1 were not supported by satellite measurements of tropospheric column. Similar to the analysis of NO_2,_ satellite measurements average over a larger area and include background or transport emissions.

A slight decrease was seen in the satellite-based SO_2_ for BG, GR, RO1, RO2, and SP2. For the rest (SP1, DE, and the UK), no clear trend could be identified.

A good correlation was at PL2, RO1, RO2, and BG, where at least there were no large discrepancies between the annual variations, and decreased emissions were, most times, supported by accompanying decreases in SO_2_ measurements. A good correlation between the two sets was also seen at the DE station, where, despite the small value of the correlation coefficient, both satellite and estimated emissions showed a similar variation of the SO_2_ content. 

A different variation was seen for emissions at GR, PL1, RO1, and RO2, which went down to 40% of their initial values in 2005, 2006, and 2007. This decrease, however, was not backed up by satellite measurements whose variation is (as for the UK) about 25% of the maximum. Something marginally similar was seen in SP2 and GR, where the decrease in emissions after 2008 was even more dramatic, going down by almost 90%. Satellite measurements partly supported, in general, the decrease in SO_2_, even if the corresponding variation was only 30%–50%. This discrepancy can be explained by possible additional SO_2_ loading seized by the satellite instrument. However, natural sources of SO_2_ cannot account for the difference, and most SO_2_ originated from coal and oil burning, unlike NO_2_. 

For the UK, the situation is completely different, since both emissions and measurements vary within the same range, but they are anticorrelated. The effects of the cold winter of 2010 and the financial and economic crisis on the SO_2_ variability were less visible than for NO_2_. This may have been related to the fact that the power plants had improved their SO_2_ filtering system, the burning system and the quality of the fuel used, e.g., this would be the case of Romanian power plants, for which the implementation of the EU directives was achieved gradually during 2007–2013 [28]. This was seen both in satellite measurements and in the emissions for stations in Romania (RO1 and RO2).

Some inconsistencies between space observations and ground emissions may also arise from the electricity demand or the energy consumption curve, which is represented by the electricity demand function of time. The space sensor can have the overpass time during high demand of electricity, which corresponds to high emissions. OMI passes over the Romanian LCP around 10–12 UTC, which corresponds to the time interval of high demand for electricity [46]. In such a case, i.e., when the overpass time of the space sensor coincides with a high demand of energy, thus of SO_2_, this may explain the fact that the satellite-based SO_2_ may overestimates the SO_2_ emissions. The reported emissions are based on daily emissions calculations while the space observations are based-on one, two, or no observation per day function of orbit. Note that the EU Directives does not impose hourly or daily limits for the emissions, the emission restrictions are quantified annually. 

**Figure 9:**
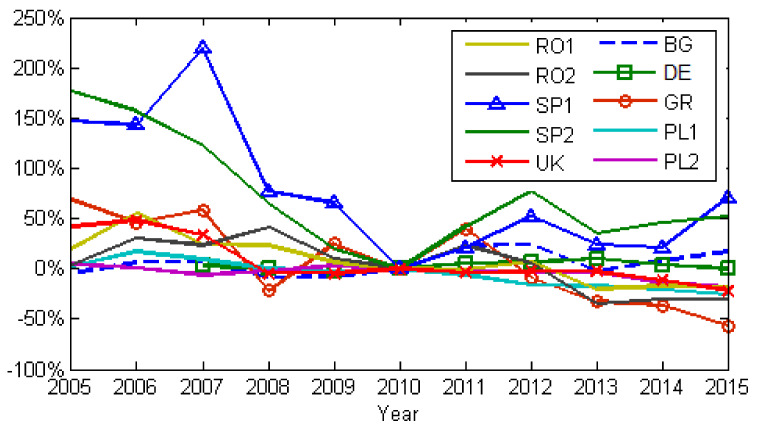
Changes in NO_x_ emissions relative to 2010, the milestone being NEC Directive.

**Figure 10:**
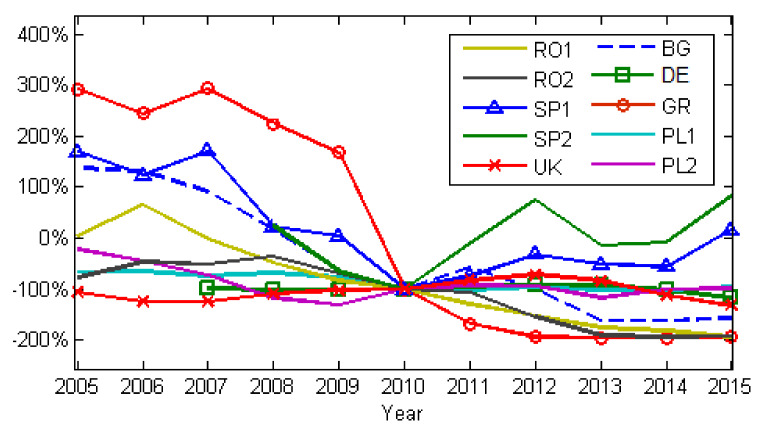
Changes in SO_2_ emissions relative to 2010, the milestone being NEC Directive.

**Figure 11:**
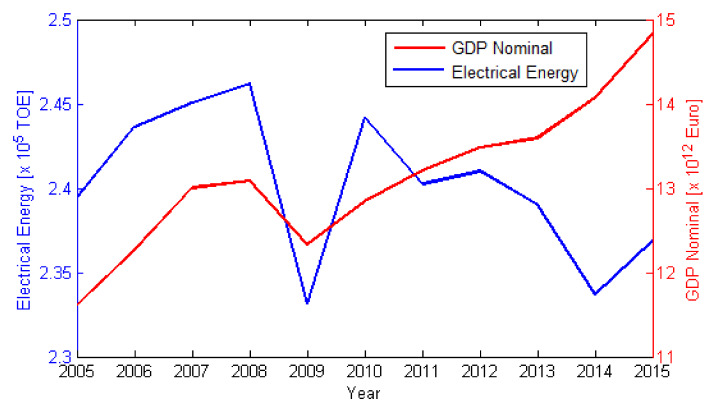
Comparison between Nominal GDP and Electrical Energy over the EU during 2005–2015, where TOE represents a tonne of oil equivalent (source: Eurostat).

Figure 9 and Figure 10 show the annual changes in emissions relative to 2010, for NOx and SO_2_. We chose 2010 because, by then, the level of national emissions, including those caused by LCPs, should have complied with the NEC Directive (2001/81/EC). According to Figure 5 and Figure 7, emissions for all LCPs presented in this work show descending trends until 2010. After 2010 a clear increasing trend for both NOx and SO_2_ emissions was observed only for power plants in Spain. The NOx and SO_2_ emissions descending trend was the result of the EU Directives, combined with the financial and economic crisis during 2007–2009. Figure 11 shows the influence of the financial and economic crisis on the evolution of the Nominal GDP (Gross Domestic Product) and electrical energy production using fossil fuels during 2005–2015 (Eurostat, https://ec.europa.eu/eurostat/data/database).

## 4. Conclusions

In this paper, we presented the variation of NO_2_ and SO_2_ atmospheric emissions attributed to ten very large power plants across Europe (EU-28), quantified by calculated emissions and by satellite observations, during 2005–2015. The main aim of this work was to study the evolution of the NO_2_ and SO_2_ amount after implementation of the LCP Directive (2001/80/EC), and the NEC Directive (2001/81/EC). Another goal of the comparison was to see to what extent changes in reported emissions to EEA by each country are supported by OMI space-based observations. We presented that OMI observations support the changes in reported emissions to EEA by the EU-28 countries. We identified most of the main drivers for the emissions decrease or increase during 2005–2015. We found that the financial and economic crisis during 2007–2009 had an important role in the emission reduction before 2010.

In general, the NO_2_ and SO_2_ emissions from LCPs in the Eastern European countries were larger than for Western European countries. During the period between 2005 and 2015, some Eastern Europe socio-political changes triggered by the accession to the European Union took place, requiring compliance with the legislation imposing pollution limits. These changes were directly reflected in the corresponding emission reductions of NO_2_ and SO_2_. The reduction in solid fuel consumption (in both Eastern and Western Europe) was balanced by the growth of gas consumption or by renewable and nuclear energy [47]. In Eastern Europe, OMI showed a substantial SO_2_ reduction in the proximity of the coal-fired power plants, because flue-gas desulfurization equipment was installed during the study period. 

The satellite observations clearly detected a decreasing tendency of NO_2_ and SO_2_ amount for the entire analyzed period. The satellite observations presented in this paper support the conclusion about the recent decline in NOx and SO_2_ emissions from power plants across Europe, where the EU policies for emission reduction played a key role in the LCP emissions reduction. Future work will focus on the impact assessment of the Industrial Emissions Directive (IED) over the LCPs presented in this study.

## Figures and Tables

**Table 1:** Information for individual power plants (source: EEA *).

Power Plant	Country	Code	Lat. (°)	Long. (°)	Average Annual Power (MWth)	Average Annual SO_2_ Emissions (T)	Average Annual NO_X_ Emissios (t)
1. TPP “Maritsa Iztok 2” TPP “Maritsa Iztok 3” Stara Zagora	Bulgaria	BG	42.25 42.05	26.13 25.62	6743	232,084	16,211
2. KW Jänschwalde, Peitz	Germany	DE	51.83	14.46	9144	21,438	19,218
3. PPC S.A.–Megalopoli I-IV	Greece	GR	37.41	22.10	2381	108,620	4543.5
4. Elektrownia “Kozienice” S.A.	Poland	PL1	51.66	21.46	7023.1	41,334	20,544
5. PGE Górnictwo i Energetyka Konwencjonalna S.A.–Oddział Elektrownia Bełchatów, Łódź Voivodeship	Poland	PL2	51.26	19.33	13170	80,789	40,217
6. S.C. Complexul Energetic Oltenia S.A., Rovinari 1-2	Romania	RO1	44.90	23.13	3512	60,166	11,937
7. S.C. Complexul Energetic Turceni S.A. 1-4	Romania	RO2	44.66	23.41	4734	72,824	14,526
8. CT LITORAL I-II, Carboneras-Almeria	Spain	SP1	36.97	−1.90	2737.3	14,929	10,453
9. Central térmica de Puentes de García Rodríguez, La Coruña (CT AS PONTES I-II-III-IV)	Spain	SP2	43.44	−7.86	3795.3	76,525	10,783
10. Drax Power Limited, Drax Power Station	United Kingdom	UK	53.73	−0.99	10145	26,094	42,954

* https://www.eea.europa.eu/data-and-maps/data/lcp-9.
